# Leishmania major Strain-Dependent Macrophage Activation Contributes to Pathogenicity in the Absence of Lymphocytes

**DOI:** 10.1128/spectrum.01126-22

**Published:** 2022-10-03

**Authors:** Jalal Alshaweesh, Risa Nakamura, Yuka Tanaka, Mizuki Hayashishita, Abu Musa, Mihoko Kikuchi, Daniel Ken Inaoka, Shinjiro Hamano

**Affiliations:** a Department of Parasitology, Institute of Tropical Medicine (NEKKEN), Nagasaki University, Nagasaki, Japan; b Graduate School of Biomedical Sciences, Doctoral Leadership Program, Nagasaki University, Nagasaki, Japan; c School of Tropical Medicine and Global Health, WISE Programme, Nagasaki University, Nagasaki, Japan; d The Joint Research Center on Tropical Diseases, Institute of Tropical Medicine (NEKKEN), Nagasaki University, Nagasaki, Japan; e School of Tropical Medicine and Global Health, Nagasaki University, Nagasaki, Japan; f Dana-Farber Cancer Institute, Harvard Medical University, Boston, Massachusetts, USA; g Department of Immunogenetics, Institute of Tropical Medicine (NEKKEN), Nagasaki University, Nagasaki, Japan; h Department of Molecular Infection Dynamics, Shionogi Global Infectious Disease Division, Institute of Tropical Medicine (NEKKEN), Nagasaki University, Nagasaki, Japan; University of Arkansas for Medical Sciences

**Keywords:** *Leishmania major*, cutaneous leishmaniasis, lymphocyte-independent pathology, strain-dependent virulence, neutrophilic inflammation, macrophage activation, TLR2

## Abstract

Infection of C57BL/6 wild-type mice with Leishmania major 5-ASKH or Friedlin strains results in relatively similar pathogenicity with self-healing lesions within weeks. Parasite clearance depends on nitric oxide production by activated macrophages in response to cytokines produced mainly by CD4^+^ Th1 cells. In contrast, C57BL/6 Rag2 knockout mice, which lack T and B lymphocytes, show distinct pathologies during infection with these strains. Despite of the similar parasite number, the 5-ASKH infection induced severe inflammation rather than the Friedlin. To determine the immunological factors behind this phenomenon, we infected C57BL/6 Rag2 knockout mice with these two strains and compared immune cell kinetics and macrophage activation status. Compared with the Friedlin strain, the 5-ASKH strain elicited increased pathology associated with the accumulation of CD11b^high^, Ly6G^high^ neutrophils by week four and increased the expression of macrophage activation markers. We then analyzed the differentially expressed transcripts in infected bone marrow-derived macrophages by RNA sequencing. It showed upregulation of multiple inflammatory transcripts, including Toll-like receptor 1/2 (TLR1/2), CD69, and CARD14, upon 5-ASKH infection. Our findings suggest that different L. major strains can trigger distinct macrophage activation, contributing to the disease outcome observed in the absence of lymphocytes but not in the presence of lymphocytes.

**IMPORTANCE** Disease manifestations of cutaneous leishmaniasis (CL) range from self-healing cutaneous lesions to chronic forms of the disease, depending on the infecting *Leishmania* sp. and host immune protection. Previous works on mouse models of CL show the distinct pathogenicity of Leishmania major strains in the absence of lymphocytes. However, the mechanisms of this pathology remain uncovered. In the trial to understand the immunological process involved in lymphocyte-independent pathology, we have found a specific induction of macrophages by different L. major strains that affect their ability to mount innate responses leading to neutrophilic pathology when lymphocytes are ablated.

## INTRODUCTION

Leishmaniasis is a neglected tropical disease caused by a protozoan parasite, the genus of *Leishmania*. It shuttles between sandfly vectors and mammalian hosts. They are obligatory intracellular parasites in mammals and proliferate as amastigotes in phagocytes, while they multiply as promastigotes in the midgut of the sandfly (1). Leishmaniasis is caused by over 20 different *Leishmania* species contributing to a wide range of clinical manifestations with various symptoms, from a self-healing cutaneous leishmaniasis (CL) to a life-threatening systemic visceral leishmaniasis (VL) (2). These heterogeneous outcomes are attributed to the interplay of various factors, including parasite infectivity, virulence, pathogenicity, host genetic factor, immune response, and vector biology (3, 4). CL is the most common form of the disease, with a total of 0.7 to 1 million CL cases per year, according to the WHO based on reports from about 100 countries (5). However, CL also varies in severity and clinical appearance despite a self-limited disease, ranging from a small, localized skin lesion (ulcerative, nodular, volcano-shaped, Zostera, and multiple) to a severe chronic disfiguring mucosal form (6, 7). Furthermore, the host conditions significantly influence the CL severity, including coinfection with HIV, immunosuppression, and malnutrition (8–10). HIV is a well-established risk factor due to the increased CL detection associated with HIV (11).

While most studies on leishmaniasis in humans are descriptive, the immune response against Leishmania has been clarified extensively using experimental models. The murine model of Leishmania major infection is the most dominant one. Disease control generally depends on developing Th1 cell-mediated immunity and interferon gamma (IFN-γ) production, activating macrophages and inducing a leishmanicidal state with nitric oxide production (12, 13). The enhanced inflammatory responses that mediate the pathogenesis are triggered by parasite factors and the host’s innate and adaptive immune responses (14). Also, parasite factors can contribute to the disease severity with the considerable variation in gene homologs and the high degree of single nucleotide polymorphisms (SNPs) among intraspecies and interspecies (15). Using a single strain in well-controlled *in vitro* or *in vivo* models, several studies documented a controversial disease outcome in mouse models lacking T/B lymphocytes. These mice were shown to have enhanced or decreased inflammation compared with the immunocompetent mice (16–21). However, the immunopathology in the absence of T/B lymphocytes has not been studied adequately.

We previously described two strains of L. major, namely, 5-ASKH and Friedlin, which showed distinct pathogenicity in the absence of lymphocytes (17). The immunological processes involved in this particular pathology remain uncovered. As it would help us understand the disease outcome in the immunocompromised patient, we used C57BL/6 Rag2^−/−^ mice that lack T/B cells and two different strains of L. major that provoke the distinct phenotypes (17).

## RESULTS

### The severe inflammation induced by 5-ASKH infection in C57BL/6 Rag2^−/−^ mice correlates with the accumulation of neutrophils.

**FIG 1:**
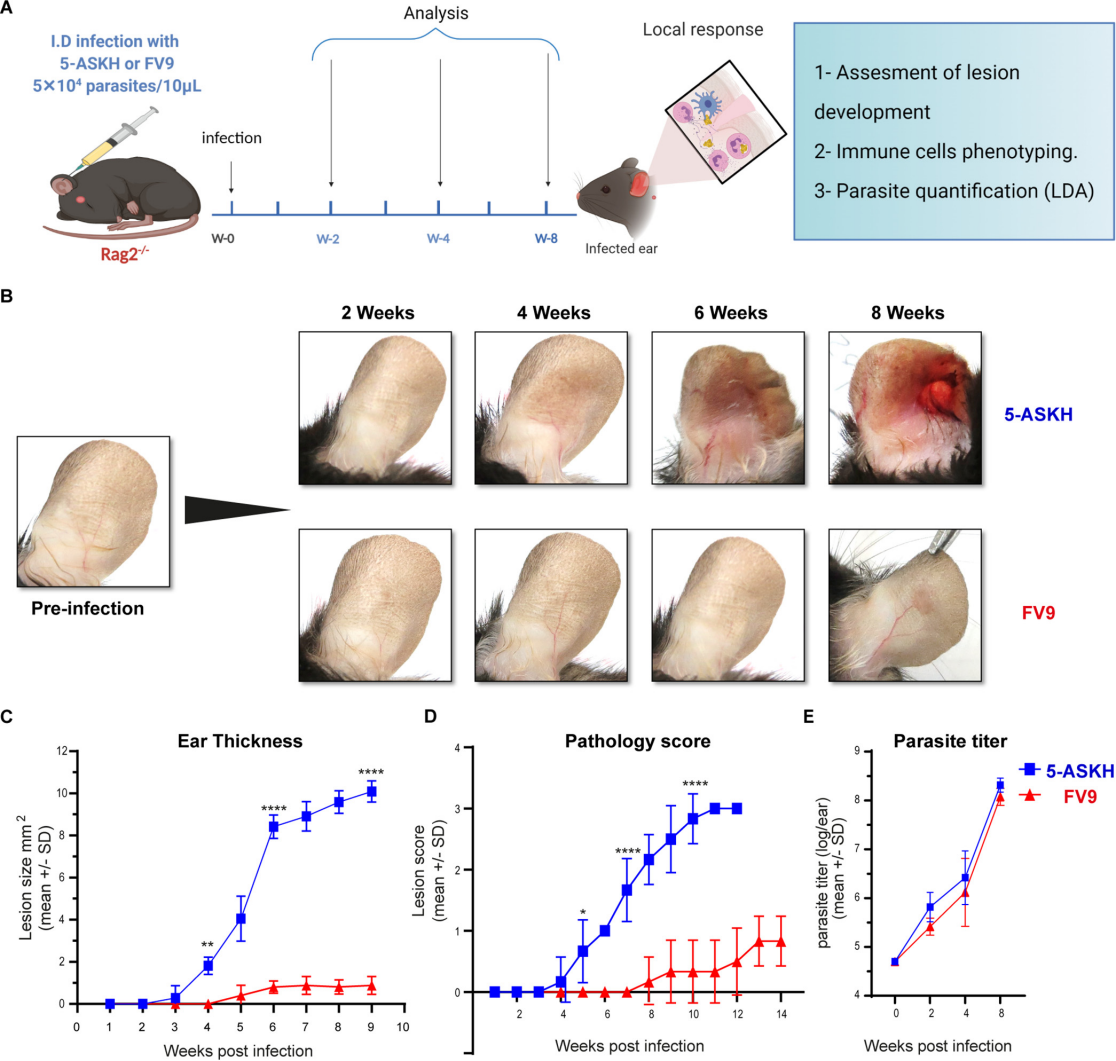
Despite having a similar parasite load, 5-ASKH produces more severe pathology in C57BL/6 Rag2^−/−^ mice than FV9. C57BL/6 Rag2^−/−^ mice were infected in the ear dermis with 5 × 10^4^ 5-ASKH or FV9 metacyclic promastigotes. (A) Illustration of the experimental design. (B) Photographs of mouse ears taken at different time points during the course of infection. (C) Lesion development as measured weekly using a caliper. (D) Pathology was scored as follows: 0, no lesion; 1, inflamed lesion; 2, ulcered lesion; and 3, eroded ear. (E) Parasite burden as determined by limiting dilution assay (LDA) in the ear lesion at 2, 4, and 8 weeks postinfection shown as log_10_ (parasite/ear). Results are shown as mean ± SD, and data are representative of 3 independent experiments with 3 to 5 mice/group. *, *P* < 0.05; **, *P* ≤ 0.01; ***, *P* ≤ 0.001; and ****, *P* ≤ 0.0001 comparing infection groups.

**FIG 2:**
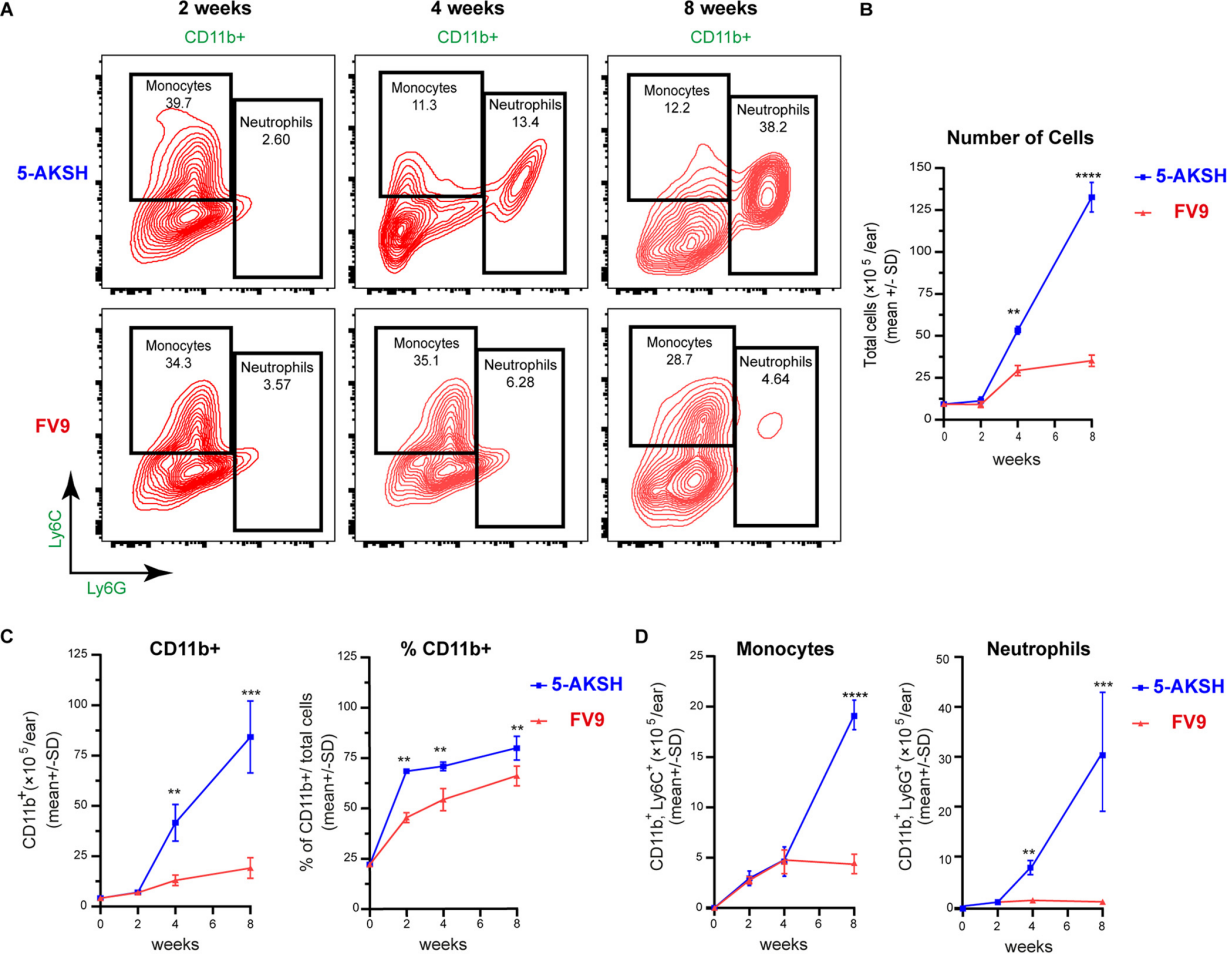
Kinetics of CD11b^+^ cell accumulation in the site of infection with the accumulation of Ly6G^+^ neutrophils in 5-ASKH infection. C57BL/6 Rag2^−/−^ mice were infected in the ear dermis with 5 × 10^4^ 5-ASKH or FV9 metacyclic promastigotes; ear tissues were processed and phenotyped by flow cytometry at 2, 4, and 8 weeks postinfection. (A) Representative dot plots of ear-derived live, CD11b^+^ myeloid cells, which were defined as Ly6G^high^ Ly6C^int^ (neutrophils), Ly6G^−^ Ly6C^high^ (monocyte), and Ly6G^−^ Ly6C^−^ (dermal macrophages and DCs). (B) The total number of cells in infected ears over the course of infection. (C) The total number/percentage of CD11b^+^ cells. (D) The total number of monocytes and neutrophils, respectively. Results are shown as mean ± SD, and data are representative of 2 independent experiments with 3 to 5 mice/group. *, *P* < 0.05; **, *P* ≤ 0.01; ***, *P* ≤ 0.001; and ****, *P* ≤ 0.0001 comparing infection groups.

First, we infected C57BL/6 Rag2^−/−^ mice, lacking T and B cells, with 5 × 10^4^ L. major parasites into the ear dermis. The mice infected with 5-ASKH exhibited lesions by 4 weeks postinfection and were characterized by severe tissue destruction with ulcer (Fig. 1B) and eventual ear loss (not shown). In contrast, FV9-infected mice gradually developed nodular lesions by 8 weeks (Fig. 1A to D). Both strains showed a similar parasite number in the infected ears (Fig. 1E). Then, we analyzed immune cell kinetics to better define the immunological events contributing to the increased local inflammation in the 5-ASKH-infected mice. Since phagocytes are the primary cells infected with *Leishmania* parasites, disease development depends on the direct interaction between *Leishmania* and macrophages (22). Therefore, we focused on the myeloid population defined by their expression of CD11b^+^ (see Fig. S3 in the supplemental material). Based on the Ly6C and Ly6G expression, we classified the live CD11b^+^ cells into the groups CD11b^+^, Ly6C^–^, Ly6G^–^ (dermal dendritic cells [DCs]/macrophages); CD11b^+^, Ly6C^int^, Ly6G^hi^ (neutrophils); and CD11b^+^, Ly6C^hi^, Ly6G^low^ (monocytes) (Fig. 2A). The total number of cells, including CD11b^+^ cells, increased slightly during the first 2 weeks of infection with both strains, with a similar proportion of neutrophils and monocytes. At 4 weeks postinfection, CD11b^+^ cells were recruited more with 5-ASKH than with FV9, which correlated with the skin lesion development (Fig. 2B and C). A dramatic increase in CD11b^+^ cells was observed at 8 weeks postinfection with 5-ASKH (Fig. 2C). The constant recruitment of Ly6G^hi^ neutrophils to the infection site primarily accounted for differences in CD11b^+^ infiltrate in mice infected with 5-ASKH, which was fewer observed in mice with FV9 (Fig. 2D). In addition, monocyte chemoattractant protein 1 (MCP-1), interleukin-6 (IL-6), and tumor necrosis factor alpha (TNF-α) were elevated in the serum of 5-ASKH-infected mice (see Fig. S4 in the supplemental material).

### Accumulated neutrophils increased the expression of CD49d and CD11c.

**FIG 3:**
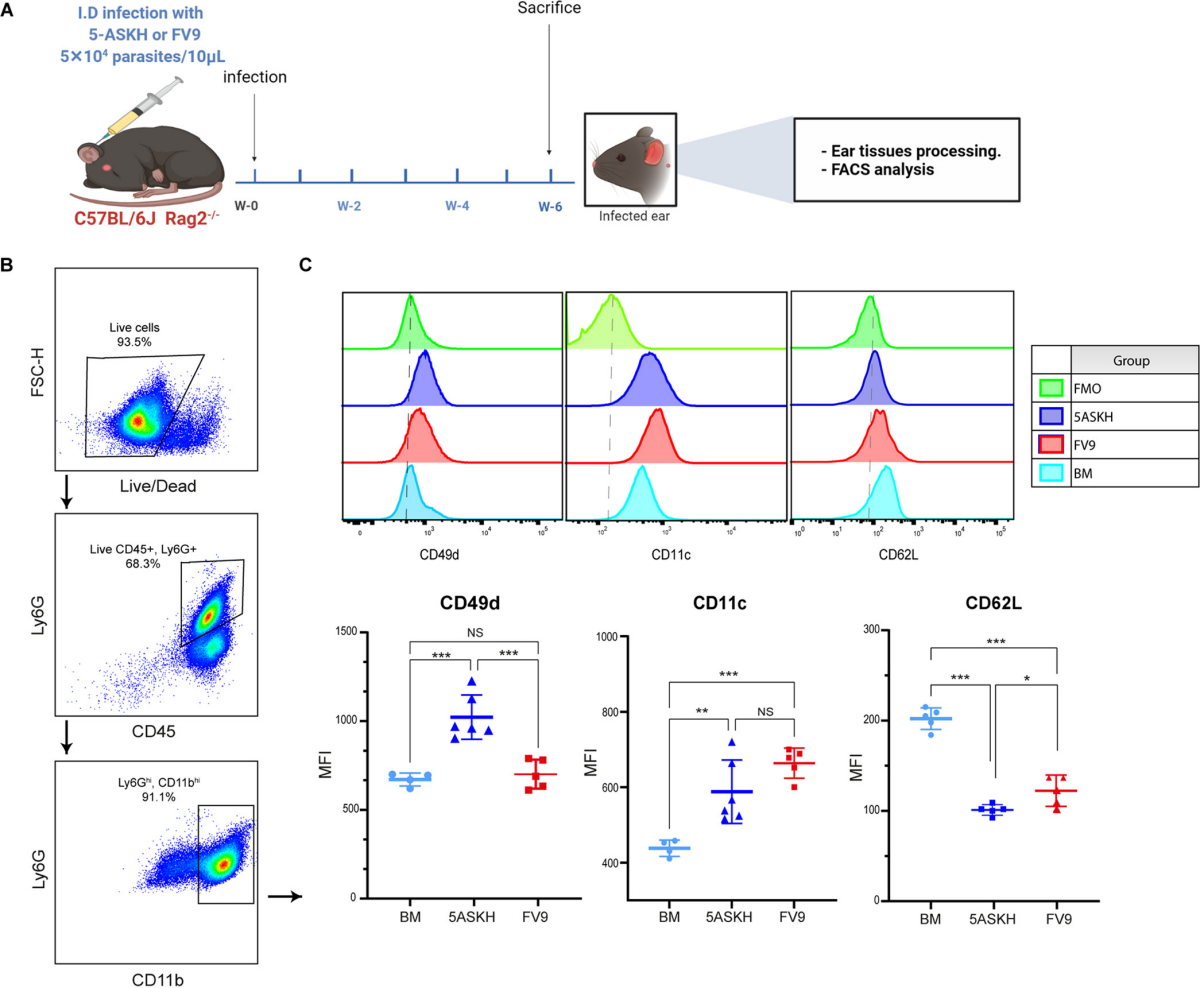
Immunophenotyping of neutrophils recruited into the skin dermis. C57BL/6 Rag2^−/−^ mice were infected in the ear dermis with 5 × 10^4^ 5-ASKH or FV9 metacyclic promastigotes, and the ear tissues were processed and phenotyped by flow cytometry at 6 weeks postinfection. (A) Illustration of the experimental design. (B) Representative dot plots of gating strategy. (C) The expression of CD11c, CD62L, and CD49d on neutrophils (CD45^+^, CD11b^high^, Ly6G^high^). Results are shown as mean ± SD, and data are representative of 2 independent experiments with 5 mice/group. *, *P* < 0.05; **, *P* ≤ 0.01; and ***, *P* ≤ 0.001, comparing infection groups. BM, bone marrow; MFI, mean fluorescence intensity.

Since immunophenotyping of CD11b^+^ infiltrates revealed a significant difference in neutrophil accumulation in the infection site, we assessed the phenotype of the accumulated neutrophils by examining the expression of adherence molecules, including L-selecting (CD62L), α4-integrin (CD49d), and β2-integrin (CD11c), which are expressed differentially on their surface under inflammatory conditions and are associated with neutrophil recruitment from the circulation (23–25). Rag2^−/−^ mice were infected with 5 × 10^4^ L. major (5-ASKH, FV9) parasites, and the expression of CD62L, CD49d, and CD11c on neutrophils was evaluated at 6 weeks postinfection (Fig. 3A). Isolated neutrophils from bone marrow (BM) of naive littermates were used as the control. Neutrophils recruited to the ear dermis showed a decreased expression of CD62L and increased CD11c expression in both infections (5-ASKH and FV9) compared with BM neutrophils. However, only neutrophils from 5-ASKH-infected mice showed an increased expression in CD49d compared with those in FV9 (Fig. 3B and C).

### 5-ASKH and FV9 infection differentially activate dermal macrophages in Rag2^−/−^ mice.

**FIG 4:**
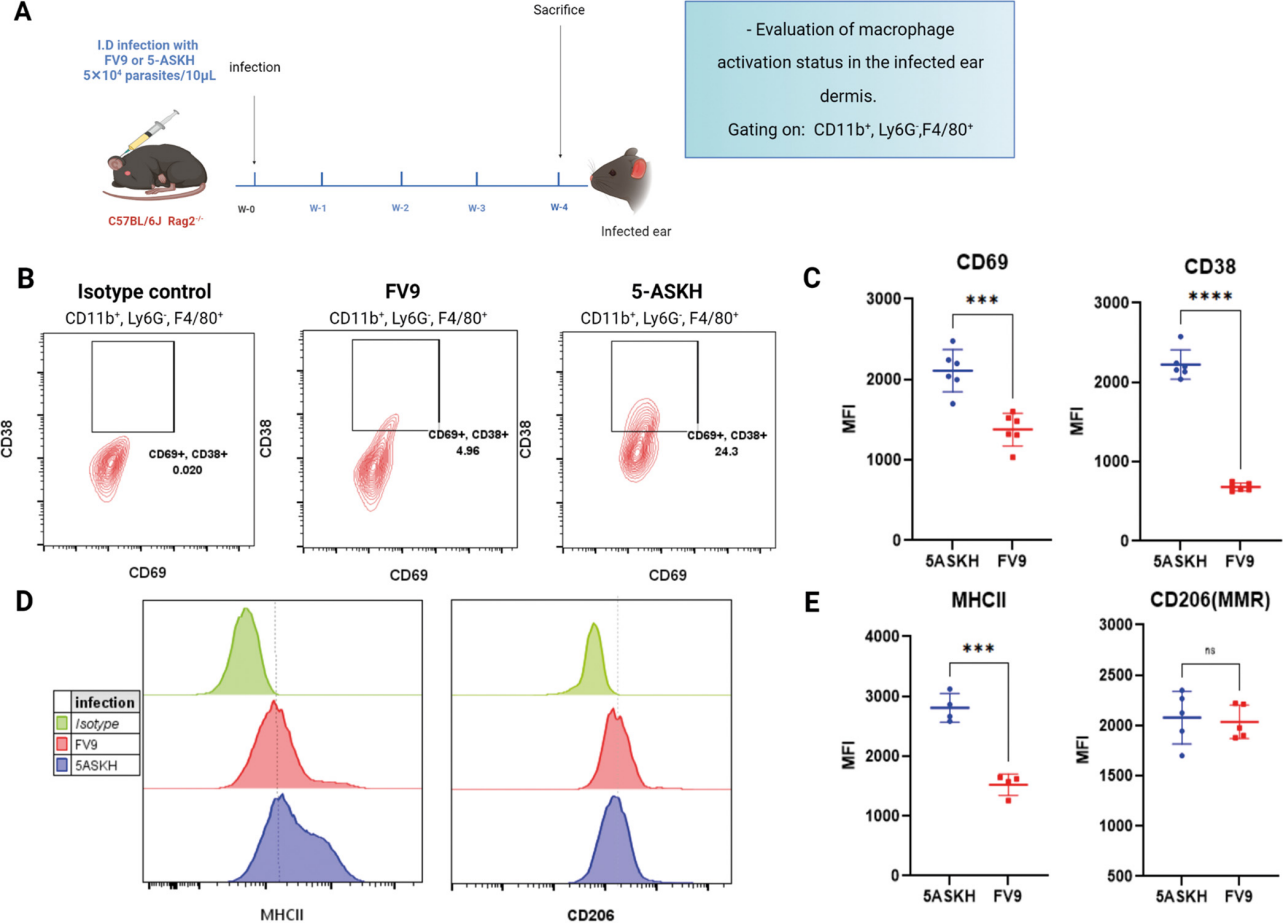
Ear dermis CD11b^+^ Ly6G^−^ F4/80^+^ macrophages upregulate the expression of CD38 and CD69 at 4 weeks after 5-ASKH infection. C57BL/6 Rag2^−/−^ mice were infected in the ear dermis with 5 × 10^4^ 5-ASKH or FV9 metacyclic promastigotes, and the ear tissues were processed and phenotyped by flow cytometry at 4 weeks postinfection. (A) Illustration of the experimental design. (B) Representative flow cytometry dot plots with CD38 and CD69 expression by CD11b^+^ Ly6G^−^ F4/80^+^ macrophages, namely, control (left), FV9 (middle), and 5-ASKH infection (right). (C) The corresponding MFI of the representative experiment. (D) Representative histogram showing MHC-II and CD206 expression by CD11b^+^ Ly6G^−^ F4/80^+^ macrophages. (E) The corresponding MFIs. Results are shown as mean ± SD, and data are representative of 3 independent experiments with 3 to 5 mice/group. ***, *P* ≤ 0.001; and ****, *P* ≤ 0.0001 comparing infection groups.

Macrophages are the primary host of *Leishmania* and play a pivotal role in inflammation, with different activation statuses linked directly with multiple inflammatory reactions (26–29). Thus, we thought the enhanced neutrophilic inflammation observed in 5-ASKH-infected mice was due to the different macrophage activation statuses induced by each L. major strain. Hence, we examined macrophage activation status at 4 weeks postinfection when we noticed the differences in pathogenesis as a symptom (Fig. 4A). We focused on the expression of the early activation marker CD69, and its upregulation is reported in multiple inflammatory diseases (30). We also examined the expression of CD38 and major histocompatibility complex class II (MHC-II). CD38 is a macrophage activation marker, and its expression is ameliorated in an M1-like phenotype and is associated with inducible nitric oxide synthase (iNOS) production (31). We gated on the CD11b^+^ Ly6G^−^ F4/80^+^ macrophages, and CD69, CD38, CD206, and MHC-II expression was assessed. In mice infected with 5-ASKH, expressions of CD69, CD38, and MHC-II were higher than in FV9. At the same time, no difference was observed in the M2 marker CD206 (MMR) (Fig. 4A to E). Thus, increased macrophage activation was observed in 5-ASKH infection, suggesting that 5-ASKH and FV9 have distinct abilities to activate macrophages.

### 5-ASKH upregulates the expression of inflammatory genes in BMDM, unlike FV9.

**FIG 5:**
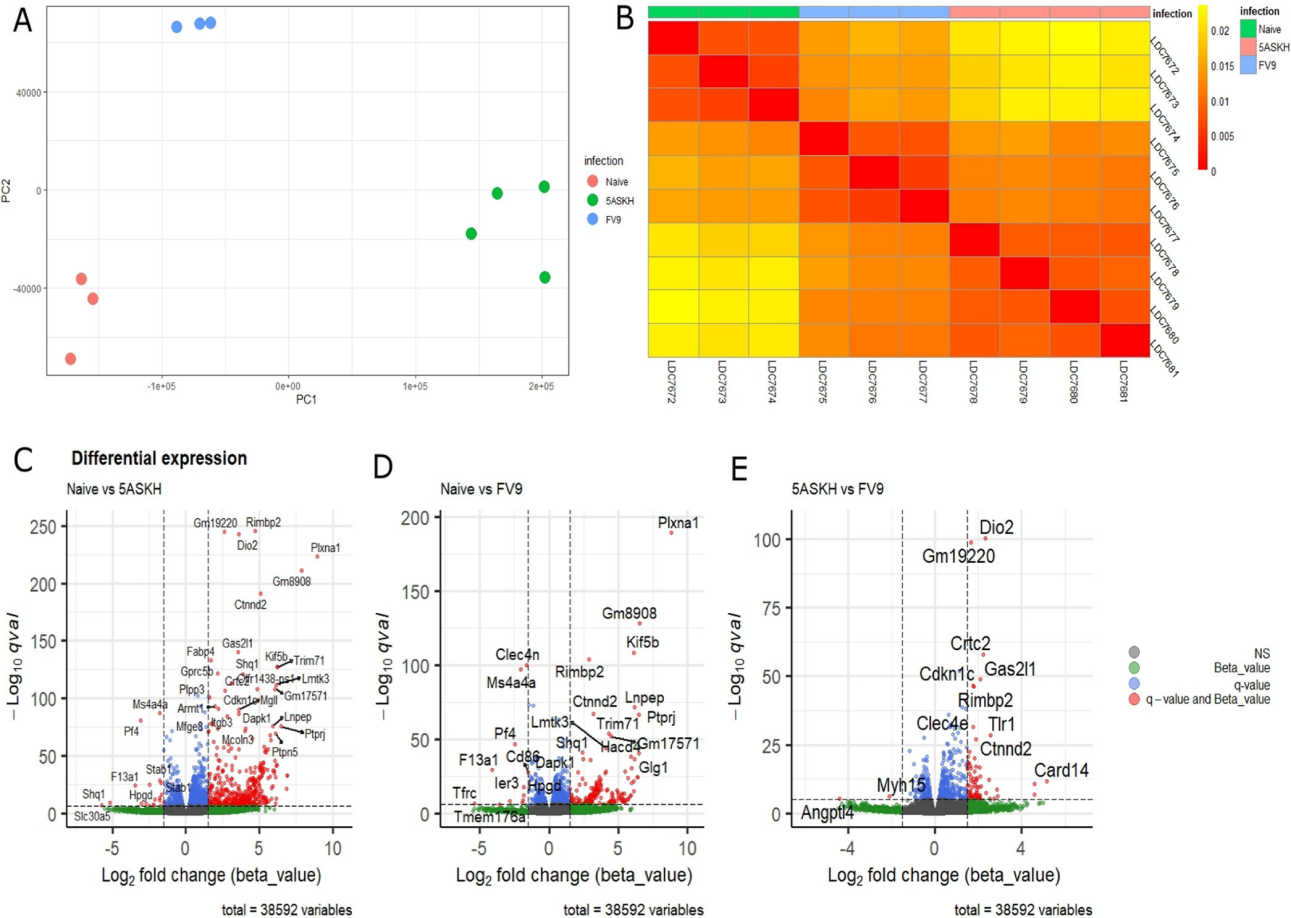
Transcriptomic profiling reveals a distinct transcripts signature of 5-ASKH or FV9 infection in BMDMs. BMDMs were cultured *in vitro* and infected for 5 days with 5-ASKH or FV9 (MOI, 10:1) or were left untreated. (A) Principal-component analysis (PCA) showing PC1 and PC2 for RNA-seq data. The samples are clearly clustered based on their infection status. The analysis was performed using the Sleuth R package, and the sample distance was computed using Jensen-Shannon divergence. (B) The corresponding sample heatmap. (C) A volcano plot of naive versus 5-ASKH infection showing log_2_ fold changes (FCs) and *q* value differentially expressed. The genes with log_2_ FC of ≥1.5 and *q* value of ≤10e^−5^ are depicted in red. (D) Naive versus FV9. (E) 5-ASKH versus FV9.

**FIG 6:**
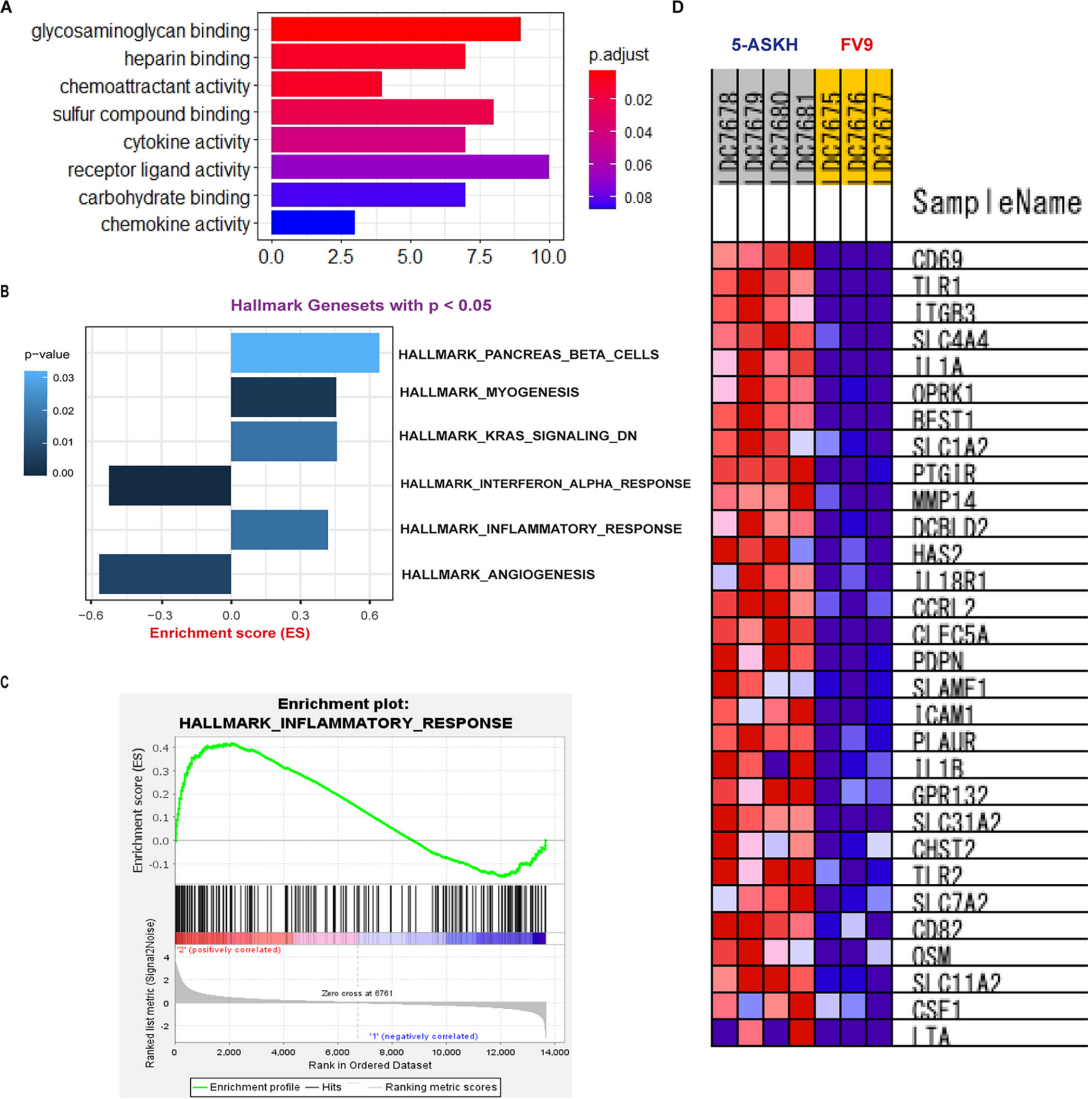
Pathway analysis of the differentially expressed transcript of 5-ASKH versus FV9. (A) Gene ontology (GO) analysis was performed using the ClusterProfiler package on the differentially expressed genes with cutoff *P* value of <0.05. The figure shows the most significant upregulated pathways with their correspondent *P* value. (B) Gene set enrichment analysis (GSEA) was mapped against the hallmark gene set; the figure shows the most significantly upregulated or downregulated gene sets with a cutoff *P* value of <0.05. (C) GSEA enrichment plot showing the hallmark inflammatory response gene set which is enriched in 5-ASKH infection with *P* value of <0.02. (D) The top 28 genes in the hallmark inflammatory response gene set enriched in 5-ASKH infection compared with those in FV9 infection.

Since 5-ASKH infection increased the activation of CD11b^+^ Ly6G^−^ F4/80^+^ macrophages, we analyzed the global gene expression of the macrophages induced by 5-ASKH and FV9 infection to identify the differentially expressed genes (DEGs). Therefore, we prepared the unprimed primary bone marrow-derived macrophages (BMDMs) and infected them *in vitro* with both strains at a ratio of 10 parasites/macrophage or left them untreated. After washing the free parasites, we incubated the BMDMs for 5 days. Then, we extracted RNA and sequenced it. The bioinformatics analysis workflow is described in Fig. S2 in the supplemental material. First, we conducted principal-component analysis (PCA) of the estimated transcript read count of the differentially infected BMDMs based on the Jensen-Shannon divergence. According to their infection status (uninfected, 5-ASKH, and FV9), replicates showed an apparent separation, confirming their distinct phenotypes (Fig. 5A and B). Next, we statistically tested the differentially expressed genes between uninfected and infected groups using the Wald test; after extracting the transcripts with more than 1.5 log_2_ fold of change and with a *q* value of <10e^−5^, we found that 5-ASKH infection upregulated 415 transcripts while FV9 infection upregulated 145 transcripts compared with uninfected BMDMs. In comparing 5-ASKH to FV9, we found only 47 upregulated transcripts in 5-ASKH with many transcripts involved in the proinflammatory response, like CARD14, Toll-like receptor 1 (TLR1), TLR2, Clec4e, and IL-1β. Among the upregulated transcripts by 5-ASKH, a CARD14 protein-coding transcript (ENSMUST00000106250) showed the highest fold of change but not the processed non-protein-coding transcript (ENSMUST00000106252) (Fig. 5E, see Fig. S6 in the supplemental material). However, only two transcripts were downregulated. Angptl4, which contributes to the anti-inflammatory response and Myh15, were downregulated in 5-ASKH infection (Fig. 5C to E; see Fig. S5 in the supplemental material). The Gene Ontology (GO) analysis of 5-ASKH versus FV9 showed the upregulation of eight pathways, including cytokine/chemokines activity (Fig. 6A). Comparing FV9-infected BMDMs with uninfected one, the transcripts associated with glycolysis, iron uptake, and oxidative stress were upregulated (see Fig. S7 in the supplemental material). At the same time, 5-ASKH infection upregulated a proinflammatory transcript and PI3K-AKT signaling pathway as shown by KEGG pathway analysis (see Fig. S8 in the supplemental material). When performing gene set enrichment analysis (GSEA) and mapping the data against the hallmark gene set, we found that four gene sets were upregulated in 5-ASKH infection compared with those in FV9. Two were downregulated with a normalized *P* value of <0.05. The hallmark inflammatory response gene set was enriched with a norm *P* value of <0.05 (Fig. 6B to D).

### Despite the similar parasite number, a lower inflammatory response was induced in MyD88^−/−^ mice compared with that of IFN-γR^−/−^ mice upon 5-ASKH infection.

**FIG 7:**
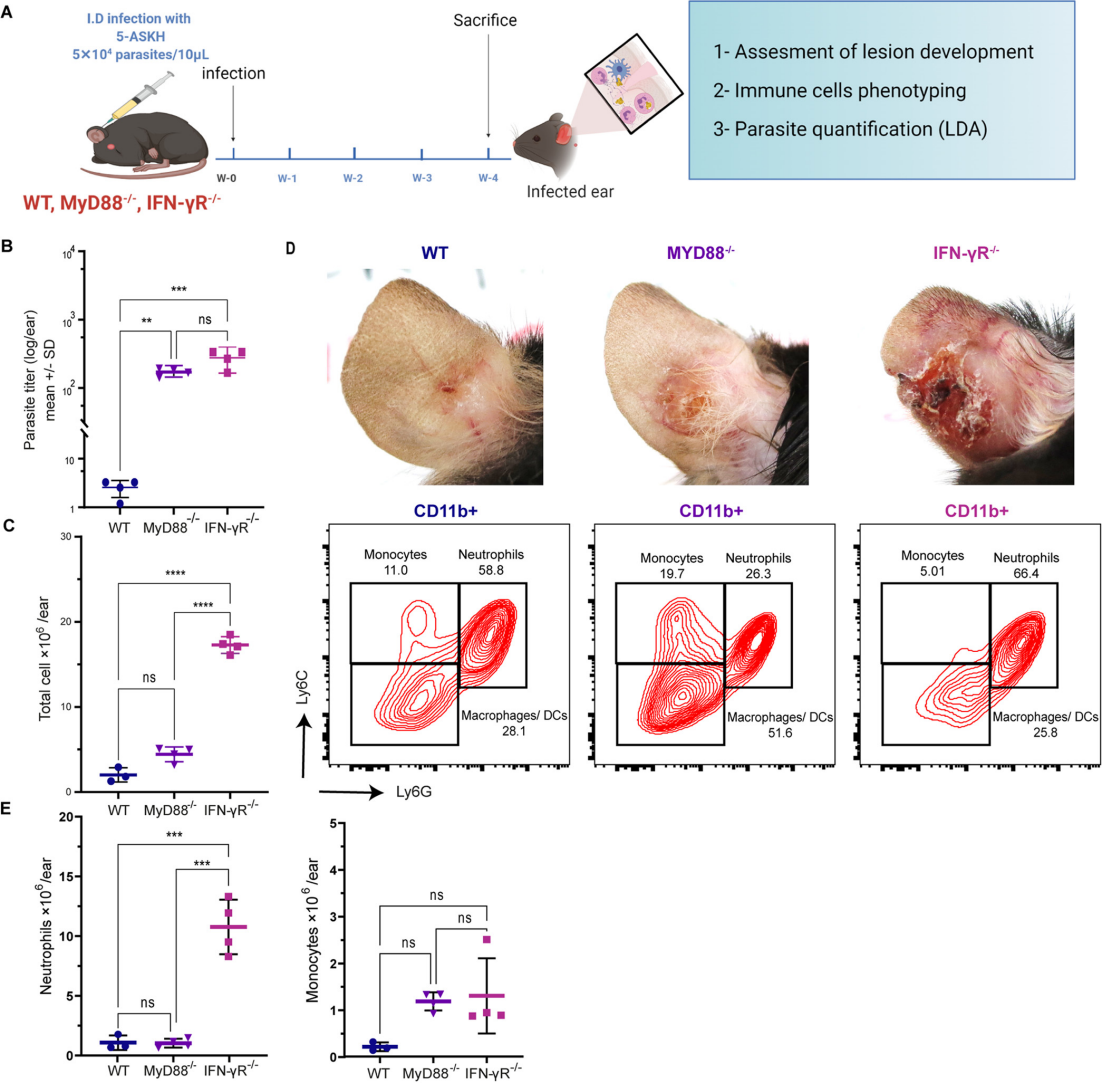
MyD88 knockout (KO) mice show less inflammation and neutrophil accumulation than IFN-γR^−/−^ mice during 5-ASKH infection. The indicated mice were infected intradermally with 5 × 10^4^ 5-ASKH metacyclic promastigotes. At 4 weeks postinfection, ear tissues were processed and phenotyped by flow cytometry, and parasite load was assessed using LDA. (A) Illustration of the experimental design. (B, C) The total number of cells and parasite burden in the ear of WT, MyD88^−/−^, IFN-γR^−/−^, and IFN-γ^−/−^ mice at 4 weeks postinfection. (D) Representative flow cytometric dot plots of ear-infiltrating CD11b^+^ myeloid cells, grouped as Ly6G^high^ Ly6C^int^ (neutrophils), Ly6G^−^ Ly6C^high^ (monocyte), and Ly6G^−^ Ly6C^−^ (CD11b^+^ Ly6G^−^ F4/80^+^ macrophages and DCs). The correspondent photographs of the ears were taken at 4 weeks postinfection. (E) The total number of neutrophils and monocytes. Results are shown as mean ± SD, and data are representative of 2 independent experiments with 3 to 4 mice/group. **, *P* ≤ 0.01; ***, *P* ≤ 0.001; and ****, *P* ≤ 0.0001 comparing infection groups.

The 5-ASKH-infected BMDMs increased the expression of inflammatory transcripts. TLR1/2 were the only pattern-recognition receptors (PRRs) significantly upregulated among them. Therefore, we thought that the TLR1/2 signaling pathway would contribute mainly to the enhanced inflammatory response in 5-ASKH infection. To investigate this hypothesis, we used MyD88^−/−^ mice, which have a defect in the downstream signaling of TLR1/2. As MyD88^−/−^ mice were expected to show a high parasite burden, we also prepared IFN-γR^−/−^ mice as a control expected to show a similar parasite number. Then, wild-type (WT), MyD88^−/−^, and IFN-γR^−/−^ mice were infected with 5 × 10^4^ parasites (Fig. 7A). At 4 weeks postinfection, MyD88^−/−^ and IFN-γR^−/−^ mice showed comparable parasite numbers. However, MyD88^−/−^ mice exhibited a lower inflammatory response than IFN-γR^−/−^ mice. The infiltrating CD11b^+^ cell number and the neutrophil proportion were much smaller in MyD88^−/−^ than those in IFN-γR^−/−^ at the site of infection (Fig. 7B to D). Comparing the phenotype of 5-ASKH and FV9 infection in IFN-γR^−/−^ mice, we showed increased neutrophil accumulation and pathology in 5-ASKH infection but not in FV9 at 4 weeks postinfection (see Fig. S9 in the supplemental material).

## DISCUSSION

This study clarified the immunological process involved in the distinct inflammatory response induced by two different strains of L. major in the absence of lymphocytes (17). 5-ASKH showed increased lesion pathology accompanied by more CD11b^+^ cells and neutrophils. At the early stage of the ear dermis lesion development, CD11b^+^ Ly6G^−^ F4/80^+^ macrophages with 5-ASKH showed increased activation compared with those of FV9, which accounted for these differences. This finding was confirmed by infecting BMDMs *in vitro* with 5-ASKH and FV9 and transcriptomic profiling using RNA sequencing (RNA-seq). Among pathogen-associated molecular pattern (PAMP recognition receptors, only TLR1/2 were upregulated in 5-ASKH-infected BMDMs). MyD88^−/−^ mice showed lower inflammation with fewer neutrophils after infection with 5-ASKH. The present findings suggest differences in macrophage activation contribute to the inflammatory process and lead to distinct lesion pathology during 5-ASKH and FV9 infection in Rag2^−/−^ mice. The impact becomes obvious when lymphocyte responses are ablated. Importantly, this finding suggests that the strain-dependent upregulation of various inflammatory transcripts, including TLR1/2, might have a possible impact on early macrophage activation and neutrophil accumulation, which would have contributed to the severe pathology in Rag2^−/−^ mice. However, further studies are required to investigate the mechanism of neutrophil accumulation and the possible contribution of TLR1/2.

Evidence from murine models of *Leishmania* infection indicates an altered pathology in immunodeficient mice due to the defective inflammatory response (19). Infection of Rag2^−/−^ mice with Leishmania donovani did not show apparent hepatosplenomegaly compared with that of WT mice, and a similar pathology was observed in HIV-positive VL patients (32, 33). In addition, skin ulceration was observed less in Rag2^−/−^ mice during CL induced by Leishmania amazonensis (20). In contrast, L. major strains cause different outcomes in Rag2^−/−^ mice, as various lesion development rates are observed in the absence of CD4^+^ T cells (18, 34, 35). Yet, it is unclear which immune cells contribute to the pathogenesis in immunocompromised mice. Here, to understand the host-parasite interactions influencing different outcomes, we have examined the kinetics of cells that mediated pathology by focusing mainly on CD11b^+^ cells and compared the transcriptomic changes in BMDMs infected with two different strains by using RNA-seq.

As reviewed previously, the roles of neutrophils are deleterious or protective in leishmaniasis depending on parasite strains and host immune response (36, 37). Our results showed that pathological neutrophilic accumulation contributes to strain-dependent tissue destruction, which is in line with the former studies showing a correlation between neutrophil accumulation and pathology (35, 38, 39). Recruited neutrophils upregulated the expression of CD49d and CD11c. It was also reported previously in neutrophils during the infection of susceptible BALB/c mice with L. major, namely, the LV39 strain (40). Upregulation of CD49d is reported in both aged neutrophils and immature neutrophils released from the bone marrow under inflammatory states (24, 41–44). Therefore, a further study is required to determine neutrophil maturation status and their altered function. Although recruitment signals were not investigated in the study, several skin chemokines are essential for the early recruitment of neutrophils to the site of infection, including CXCL2 (MIP-2) produced by monocyte/macrophages, CXCL1 produced by keratinocytes, and lipopolysaccharide-induced LIX (CXCL5) (45, 46). IL-17A is the main contributor to secondary neutrophil recruitment, and its pathological role during *Leishmania* infection was described previously (47, 48). IL-17A is produced by RORγ^+^ type 3 innate lymphocytes (ILC3), γδ T cells, natural killer (NK) cells, NKT cells, and Th17 cells. Its functions are controlled excellently by tuning balance with IFN-γ as reviewed (49). Since Rag2^−/−^ mice lack conventional T/B cells but not NK cells or ILC3, these cells may be involved in the 5-ASKH-mediated neutrophil accumulation. The pathological role of IL-17A-producing ILCs in L. major infection was described recently by Singh et al. (35). Similar to our observation, infection of Rag2^−/−^ mice with the Friedlin strain showed minimal pathology. However, it can be enhanced by skin microbiota due to the contribution of IL-17A-producing ILCs (35). In Mycobacterium tuberculosis infection, the accumulation of neutrophils is also associated with the increased pathology in Rag2^−/−^ mice. In the absence of T cell-derived IFN-γ, the balance of IL-17A/IFN-γ and the function of neutrophils may be altered (44). Therefore, further studies are warranted to investigate factors promoting neutrophil recruitment.

Macrophages are the primary host for the *Leishmania* parasite and the principal cells to trigger inflammation (12, 29). In the 5-ASKH-infected ear dermis, macrophages increased the expression of CD38 and CD69 markers. Both are proinflammatory markers, and their expression is induced robustly under inflammatory conditions (30, 31, 50). Although it has been reported that L. major can promote the M2 macrophage to support their growth (43, 44), no significant differences were observed in CD206 expression between 5-ASKH and FV9, the markers of the M2 macrophage. Collectively, these data suggest that 5-ASKH activates CD11b^+^ Ly6G^−^ F4/80^+^ macrophages rather than FV9, which seems to initiate the neutrophil recruitment process. However, we cannot rule out the contribution of other skin-resident cells, such as keratinocytes and Langerhans cells.

To our knowledge, very few studies have identified the strain-mediated transcriptomics changes in infected macrophages using RNA-seq. The differential gene expression was described recently between virulent (vAG83) and avirulent (nvAG83) L. donovani strains in peritoneal macrophages (51). In the study, the virulent L. donovani strain increased the expression of anti-inflammatory cytokines. In contrast, several parasite-specific virulence and survival factors were upregulated in the amastigote of the virulent strain (51). Strain-mediated induction of differential gene expression (DGE) was also reported recently in canine macrophages infected with Leishmania infantum (52). Several DEGs are involved in the inflammatory response, cytokine, and chemokine activity. We show using RNA-seq that both strains upregulated transcripts associated with pro- and anti-inflammatory processes, glycolysis, iron uptake, and oxidative stress. The 5-ASKH strain upregulated more proinflammatory transcripts and upregulated the PI3K-AKT signaling pathway compared with the FV9. Among TLRs that can recognize the PAMPs, only TLR1/2 is upregulated in 5-ASKH infection compared with FV9, suggesting its possible contribution to this phenotype. The upregulation of TLR2 by 5-ASKH and LV39 L. major strains, which share the exact geographic origin, was described previously (39, 53, 54). The importance of TLR2 for inducing an inflammatory response and pathology is well documented (39, 55, 56). In 5-ASKH infection, MyD88^−/−^ mice developed significantly smaller lesions with fewer neutrophil recruitment than IFN-γR^−/−^ mice, together with the equivalent parasite number, indicating critical roles of MyD88 signaling in lesion development. Compared with WT mice, MyD88^−/−^ showed a significantly increased parasite number with a slight increase in pathology with no significant difference in neutrophil recruitment. The results suggest that MyD88 is involved in the protective immune response and pathological neutrophil recruitment, aligning with a previous report that *Leishmania*-infected MyD88^−/−^ mice showed less pathology, IL-17 production, and neutrophil accumulation but a significant increase in parasite number (57, 58). In FV9 infection, IFN-γR^−/−^ showed a comparable parasite number with those infected with 5-ASKH. Still, significantly smaller lesion size and fewer neutrophils than those with 5-ASKH, reiterating distinct pathology, are not due to the number of L. major parasites between 5-ASKH- and FV9-infected mice. Neutrophils in MyD88^−/−^ mice reportedly showed a decreased count and defective recruitment (59, 60). Thus, we expect MyD88^−/−^ mice infected with FV9 to show a lesion size equivalent to that observed in FV9-infected IFN-γR^−/−^ mice or smaller (Fig. S9).

Our data suggest that impaired TLR1/2 signaling in MyD88^−/−^ mice may have contributed to this phenotype observed during 5-ASKH infection. However, currently, we cannot rule out the impact of MyD88 deficiency on other TLR-independent pathways, including IL-1 and IL-18 (61). Therefore, further studies are required to confirm this hypothesis. TLR2 is expressed in a variety of cells in the skin, including Langerhans cells, keratinocytes, and resident and trafficking myeloid cells (62, 63). Activation of TLR2 in the skin increases the expression of multiple cytokines/chemokines through NF-κB downstream signaling (64). This list includes TNF-α, IL-1, IL-6, IL-12, IL-8, and MIP2 which impact the inflammatory response and neutrophil accumulation (65–67). These data align with a previous report showing that blocking TLR2 signaling suppresses the proinflammatory response with no impact on L. major load (68). Also, TLR2^−/−^ mice infected with L. infantum showed a lower inflammatory response with decreased neutrophil recruitment in the spleen and liver (56). A similar finding was reported in the L. donovani and L. major LV39 strain (39, 69). Furthermore, TLR2, S100A9, and CXCL-2 are essential for neutrophil recruitment in both sterile and nonsterile liver inflammatory responses (70). In contrast, blockage of both TLR2 and TLR4 suppressed the proinflammatory response and parasite load in monocytes/macrophages collected from human cutaneous leishmaniasis caused by Leishmania braziliensis (55).

*Leishmania* lipophosphoglycans (LPGs) are reported to be the primary ligand of TLR2 (40, 71, 72). Furthermore, LPGs are the main virulence factor with multiple strain-specific polymorphisms in the Old world (73, 74). Therefore, our data show the distinct ability of *Leishmania* strains to induce TLR1/2 signaling and expression, which might be due to interspecies polymorphisms in LPGs that could have affected their binding and activation of TLR2.

We show for the first time a strain-dependent upregulation of the CARD14 transcript in *Leishmania*-infected BMDMs. The involvement of CARD14 in a variety of inflammatory skin disorders reported as the gain-of-function mutations is associated with psoriasis in humans and C57BL/6 mice (75). However, CARD14/CARMA2 is expressed mainly in epithelial cells and keratinocytes (76). Therefore, further study is required to investigate the strain-dependent induction of CARD14 in keratinocytes and its role in *Leishmania* pathogenesis.

Differences in strains likely pertain to some of their ability to activate macrophages and promote the innate immune response differentially. However, the parasite factors contributing to this process are not well understood. Therefore, it needs to be further investigated together with the underlying process of neutrophil accumulation. Still, given that many factors can be produced by various cell types and contribute to neutrophil recruitment, it would be informative to study the role of IL-17A-producing innate lymphocytes, such as group 3 innate lymphoid cells (ILC3) and nonhematopoietic cells, such as keratinocytes, alongside comparing neutrophil’s function induced by the strains. Also, our findings cannot fully explain the role of TLR2. Thus, further studies using Rag2 and TLR2 double-knockout mice are required to confirm the involvement of TLR2 signaling in inducing a neutrophilic inflammatory response in the absence of lymphocytes.

In conclusion, our findings show that the specific induction of macrophage activation by different L. major strain affects their ability to mount innate responses, leading to the accumulation of neutrophils and severe pathology when lymphocytes are ablated. This information opens avenues for potential new targets of immune-based therapies in immunocompromised hosts.

## MATERIALS AND METHODS

### Ethical statement.

The committee at Nagasaki University approved the study protocol for ethics on animal experiments (approval numbers 1505181226, 1505181227, 2004271624, and 2004271625) and on recombinant DNA experiments (1403041262 and 1902201550). All studies were conducted under the guidelines for animal experiments at Nagasaki University and according to Japanese law for Humane Treatment and Management of Animals (law no. 105 dated 19 October 1973, modified on 2 June 2006).

### Mice.

C57BL/6 Rag2^−/−^ (B6(Cg)-Rag2tm1.1Cgn/J) mice were purchased from Jackson Laboratory. C57BL/6 WT, IFN-γ receptor^−/−^ (IFN-γR^−/−^), MyD88^−/−^ mice on a C57BL/6 background have been maintained at Nagasaki University. Under pathogen-free conditions, all experimental mice were kept at the Animal Research Center, Nagasaki University. Six- to 8-week-old female mice were used in all experiments.

### Parasites and intradermal inoculation.

Two WHO reference strains of L. major, including Friedlin clone V9 strain (MHOM/IL/80/Friedlin) and MHOM/SU/73/5-ASKH, here referred to as FV9 and 5-ASKH, respectively, were used in this study. Both strains were passaged routinely into the footpad of BALB/c mice to maintain their virulence. Amastigotes isolated from the popliteal lymph nodes of the infected BALB/c mice were grown in the M199 medium. Promastigotes were maintained at 26°C in M199 medium (Gibco; pH 7.4) supplemented with 20% heat-inactivated fetal calf serum (FCS), 5 mg/L hemin 40 mM HEPES, 0.1 mM adenine, 1 mg/L biotin, 1 mg/L biopterin, 50 μg/mL streptomycin, and 50 U/mL penicillin. Promastigotes with less than five *in vitro* passages were used for infection studies. The infectious metacyclic promastigotes were harvested from the stationary-phase culture (4 to 5 days) using Ficoll density gradient centrifugation (77). Mice were infected intradermally (i.d.) with 5 × 10^4^ parasites/10 μL Hank’s balanced salt solution (HBSS) into the ear dermis. Lesion development was monitored weekly, and ear thickness/swelling was measured using a digital caliper. The pathology score was determined based on the macroscopic visualization of the infected skin and scored as follows: 0, no lesion; 1, inflamed lesion; 2, ulcered lesion; and 3, eroded ear.

### Processing of ears and limiting dilution assay (LDA).

Ear tissues were processed enzymatically to obtain a single-cell suspension for the limiting dilution assay and flow cytometry (78). Briefly, ears were cut off and disinfected by placing them in 70% ethanol for 3 to 5 min. Later, ear sheets were separated and placed into 2 mL of the enzymatic mixture (Dulbecco’s modified Eagle’s medium [DMEM] containing 50 μg/mL streptomycin, 50 U/mL penicillin, 50 μg/mL DNase I [Roche], and 160 μg/mL Liberase TL [Roche]). Next, ears were placed with the dermal side down and incubated for 2 h under shaking conditions at 37°C. After incubation, the enzymatic activity was inhibited with 10 mM EDTA. Next, the digested tissues were passed through a 40-μm cell strainer using a syringe plunger and washed twice with phosphate-buffered saline (PBS) with 5% FCS. Finally, cells were resuspended in 1 mL of M199 media for limited dilution or fluorescence-activated cell sorter (FACS) buffer for flow cytometric analysis.

### Flow cytometric analysis.

The ear single-cell suspension was washed two times with PBS. First, cells were adjusted to a concentration of 1 × 10^6^ in 200 μL PBS and stained with Zombie fixable dyes for 20 min at room temperature. Later, cells were washed with 1 mL of FACS buffer, resuspended in 100 μL of FACS buffer, and incubated with Fc receptor-blocking antibodies for 15 min at 4°C followed by surface staining with conjugated antibodies for 25 min at 4°C. The complete list of antibodies used in this study is described in Table S1 in the supplemental material. Stained cells were washed with FACS buffer and filtered through 30-μm filters, and data were acquired with the BD FACSCelesta instrument. Data were analyzed using FlowJo v10.8 Software (BD Life Sciences) (79).

### Cytometric bead array (CBA).

Whole blood from mice infected for 8 weeks was collected in lithium heparin blood collection tubes (Microtainers; BD Bioscience). Tubes were centrifuged at 2,000 rpm for 10 min, and serum was stored at −80°C until measurement. According to the manufacturer’s instructions, cytokine quantification was done using the cytometric bead array (CBA) mouse inflammatory kit (BD Bioscience). Data were acquired with the BD FACSCelesta instrument and analyzed with CBA Plugin v4.1 in FlowJo.

### Differentiation of bone marrow-derived macrophages (BMDMs).

As described previously, bone marrow-derived macrophages (BMDMs) were prepared from 6- to 8-week-old C57BL/6 Rag2^−/−^ mice (80, 81). Briefly, femurs and tibia were collected under aseptic conditions and separated from the connective muscles. Bone marrow was flushed with RPMI 1640 using a syringe, centrifuged at 400 × *g* for 5 min at 4°C, and filtered through a 70-μm cell strainer. After red blood cells (RBCs) were lysed, the bone marrow cells were resuspended in macrophage differentiation media (R20/30), which contains DMEM supplemented with 20% fetal calf serum (FCS), 100 U/mL penicillin, 100 μg/mL streptomycin, and 30% L929 cell-conditioned medium as a source of macrophage colony-stimulating factor (M-CSF). Cells were cultured overnight in a tissue-treated 75-cm^2^ flask (Corning). Later, the floating bone marrow cells were collected, counted, seeded into 100-mm untreated sterile petri dishes at 3 × 10^6^ cells/10 mL of R20/30, and incubated at 37°C in a CO_2_ incubator. Four days after collection, an additional 5 mL of R20/30 containing 55 μM β-mercaptoethanol (BME) was added, and cells were incubated for 3 days. On day 7, BMDMs were detached using 5 mM EDTA in PBS. The BMDM phenotype was confirmed using flow cytometry, based on their expression of CD11b and F4/80 (over 94%, see Fig. S1 in the supplemental material). Once we confirmed the phenotype, BMDMs were plated onto 6-well plates at 1 × 10^6^ cells/well in DMEM containing 10% FCS and allowed to attach by incubating them overnight. Later, the cells were washed and infected with 5-ASKH or FV9 stationary-phase parasite at a multiplicity of infection (MOI) of 10:1. Free parasites were washed out 12 h after infection, and infectivity was confirmed in separated wells using NucBlue live cell stain.

### RNA extraction, library preparation, and sequencing.

Infected BMDMs were lysed with TRIzol reagent (Invitrogen), and RNA was extracted following the manufacturer’s protocol. RNA quantification was done using Nanodrop 2000 and Qubit (Invitrogen) instruments, and quality was checked by determining the RNA integrity number (RIN) using an Agilent 4200 Tapestation system (RIN, >9.5). The NEBNext poly(A) mRNA magnetic isolation kit isolated the poly(A)-tailed mRNA from the total RNA. Library preparation was performed using the NEBNext UltraRMII directional RNA library preparation kit. The libraries were sequenced on an Illumina Novaseq 6000 to produce 150-bp paired-end reads with 6 Gb per sample.

### RNA-seq data analysis and visualization.

An analysis of the RNA-seq data was done as follows: the quality of the raw FASTQ files was checked using FastQC v0.11.9 according to the Phred quality score (82). Adapter trimming was performed using Trimmomatic v0.39 (83). Later, the trimmed reads were aligned to the mouse reference transcriptome (GRCm39, release 105), downloaded from Ensembl, and reads were quantified using Kallisto pseudoaligner v0.46.2 (84). The quality was checked using MultiQC v1.12. Kallisto abundance files were analyzed using R statistical computing environment v4.1.0 with Rstudio and Bioconductor v3.13 installed (85). Filteration, normalization, and multiple hypothesis testing were done using Sleuth package v0.30.0 (86). Mouse gene annotation was retrieved from Ensembl using biomaRt package v2.50.2 (87). Graphs were modified using EnhancedVolcano, pheatmap, and ggplot2 (88–90). For gene set enrichment analysis (GSEA), transcript-level counts were summarized to gene level using the makeLinkedTxome argument in Tximeta (91). The gene level-summarized experiment was filtered and normalized using DESeq2 (92). The analysis was done using the GSEA software v4.2.1 (Broad Institute, San Diego, CA) by mapping the expression set against the hallmark gene set (93). Pathway functional analysis and visualization were performed on LFCshrinked (ashr) DESeq2 results using ClusterProfiler package and visualized with “enrichplot” package (94, 95). KEGG pathway analysis was done using Pathview R package (96). We used the Unix distribution of the programs downloaded and managed using Anaconda (see Fig. S2 in the supplemental material).

### Software and statistical analysis.

All data were presented as mean ± SD. Two-tailed unpaired Student’s *t* test with Welch’s correction or one-way analysis of variance (ANOVA) with Tukey’s multiple comparisons were used for significance. Data analyses were performed using GraphPad Prism v9.3.1. *P* values are represented as follows: not significant (ns), *P* > 0.05; *, *P* ≤ 0.05; **, *P* ≤ 0.01; ***, *P* ≤ 0.001; and ****, *P* ≤ 0.0001. R statistical computing environment v4.1.0 was used for RNA-seq data analysis. As mentioned in the legends, the Wald test with false discovery rate (FDR) was used to determine the differentially expressed genes among groups. Gene set enrichment analysis was done using GSEA software v4.2.1 (Broad Institute, San Diego, CA). Illustrations were created with BioRender.com.

### Data availability.

The RNA-seq raw read data presented in this study can be found online at the SRA database under the BioProject number PRJNA814809.
