# Supplementary material for: Leishmania major Strain-Dependent Macrophage Activation Contributes to Pathogenicity in the Absence of Lymphocytes
Source: Microbiol Spectr. 2022 Oct 3;10(5):e01126-22. doi: 10.1128/spectrum.01126-22 (PMC9603372; doi:10.1128/spectrum.01126-22)
Supplement: Supplemental file 1 — Fig. S1 to S9 and Table S1. Download spectrum.01126-22-s0001.pdf, PDF file, 1.8 MB [file spectrum.01126-22-s0001.pdf]

Supplementary data

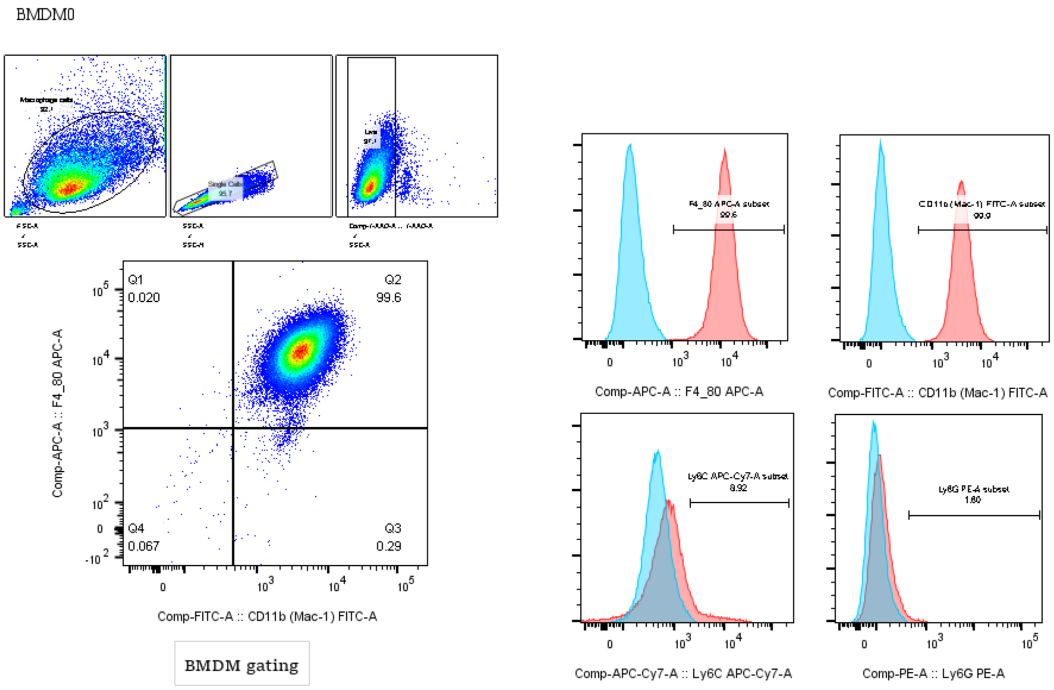

Figure S1: Bone marrow-derived macrophages phenotyping by flow cytometry. More than 96% of bone marrow-derived cells were identified as macrophages based on F4/80 and CD11b(Mac-1) expression after 7 days of culture

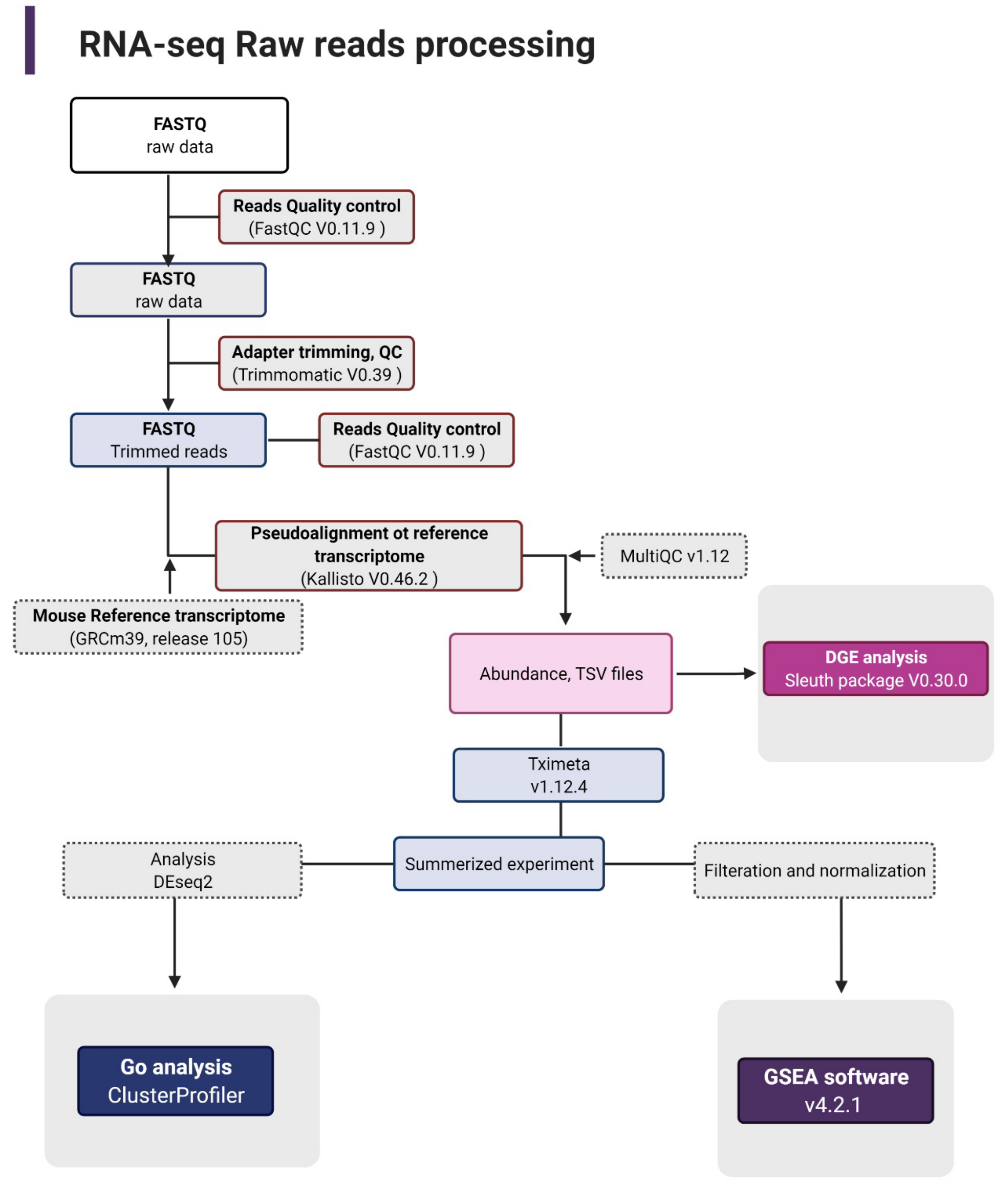

Figure S2: A flow chart showing the main RNA sequencing data processing steps Created by biorender.com.

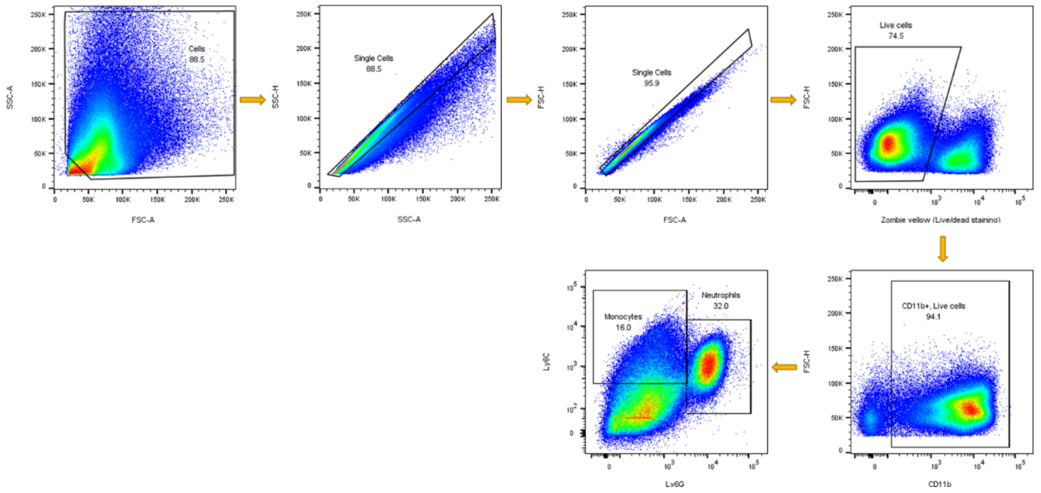

Figure S3: Flow cytometric analysis of ear dermis cells (gating strategy). C57BL/6 Rag2$^{-/-}$ mice were infected in the ear dermis with 5 × 10^4 5-ASKH or FV9 metacyclic promastigotes, ear tissues were processed and phenotyped by Flow cytometry. After doublet and dead cells discrimination, we gated on CD11b$^+$ Cells which were defined as Ly6G^{high} Ly6C^{int} (neutrophils), Ly6G$^-$ Ly6C^{high} (monocyte), Ly6G$^-$ Ly6C$^-$ (resident macrophages and DCs).

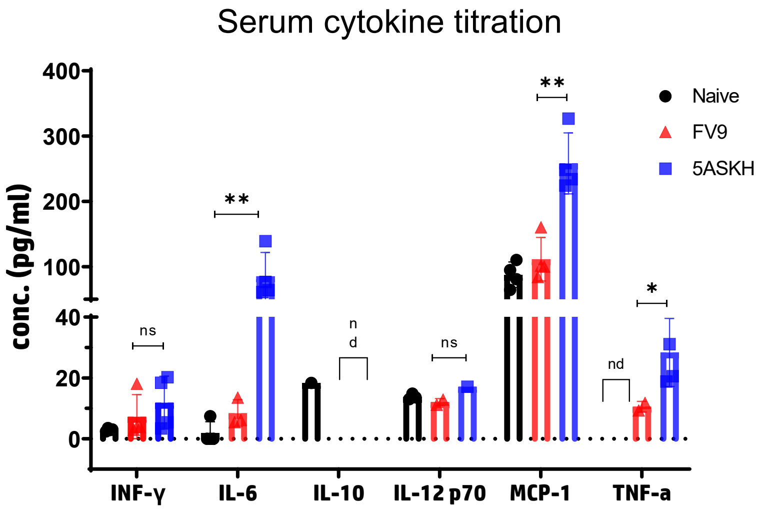

Figure S4: Quantification of inflammatory cytokines in serum samples. Rag2$^{-/-}$ mice were infected I.D. with 5 × 10^4 5-ASKH or FV9, and serum was collected at 8-weeks post-infection. Each cytokine was measured by the BD cytometric beads array (CBA) mouse inflammatory cytokine kit. In addition, unpaired comparisons of 5ASKH vs FV9 infected mice was performed using student t-test (n = 4). The samples below the detection limit were excluded from the analysis.

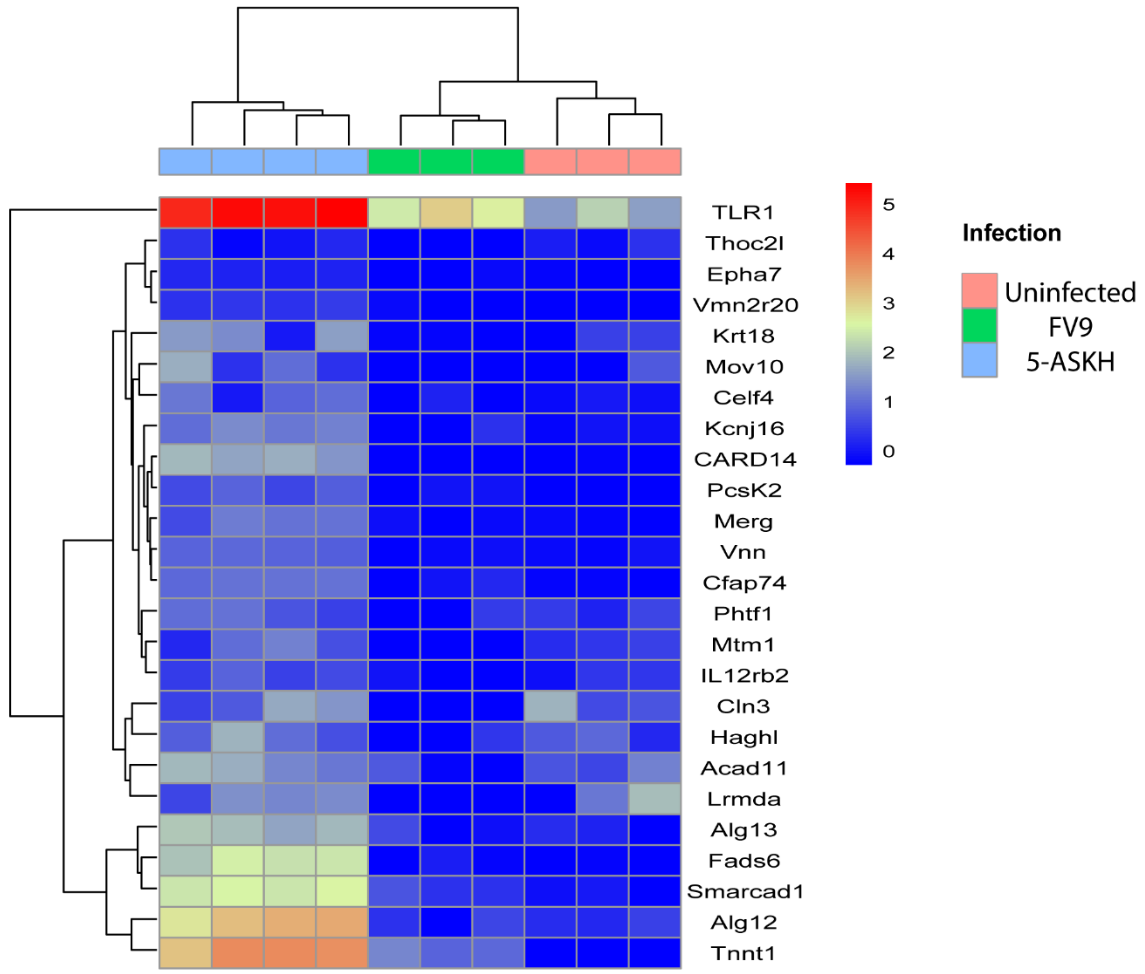

Figure S5: Samples heatmap of the differentially expressed genes. BMDM were cultured *in vitro* and infected for 5-days with 5-ASKH, FV9 (MOI; 10:1), or left untreated. The heatmap shows the top 25 differentially expressed genes log2 FC ≥ 1.5 and qvalue ≤$10e^{-5}$.

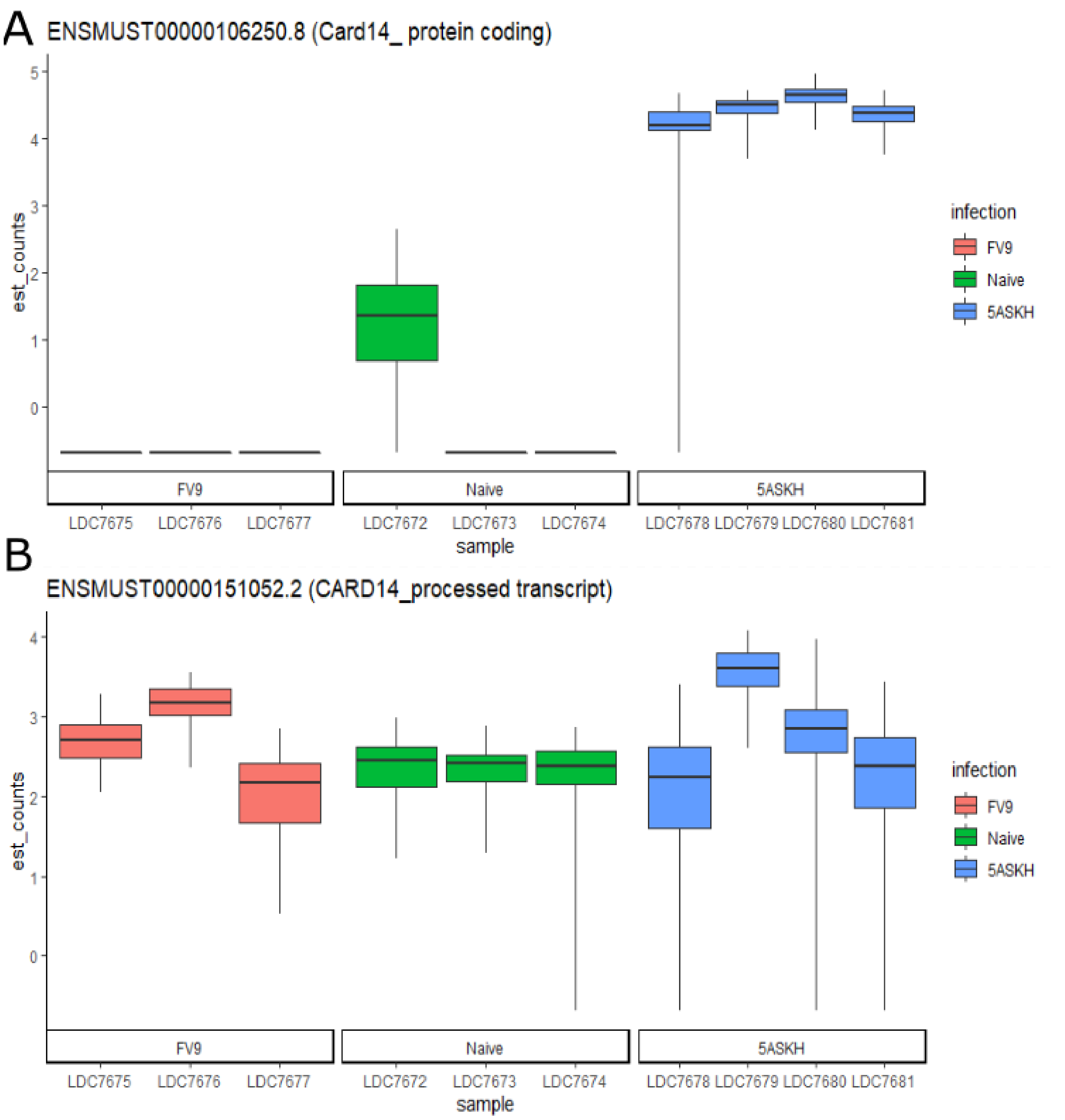

Figure S6: bootstrap plotting of CARD14 transcript generated by Sleuth R package. **(A)** boxplot of CARD14 protein-coding transcript that is upregulated in 5-ASKH, the bootstraps show that the differences are due to biological variations rather than technical variations. **(B)** The corresponding bootstraps of the unprocessed CARD14 transcript.

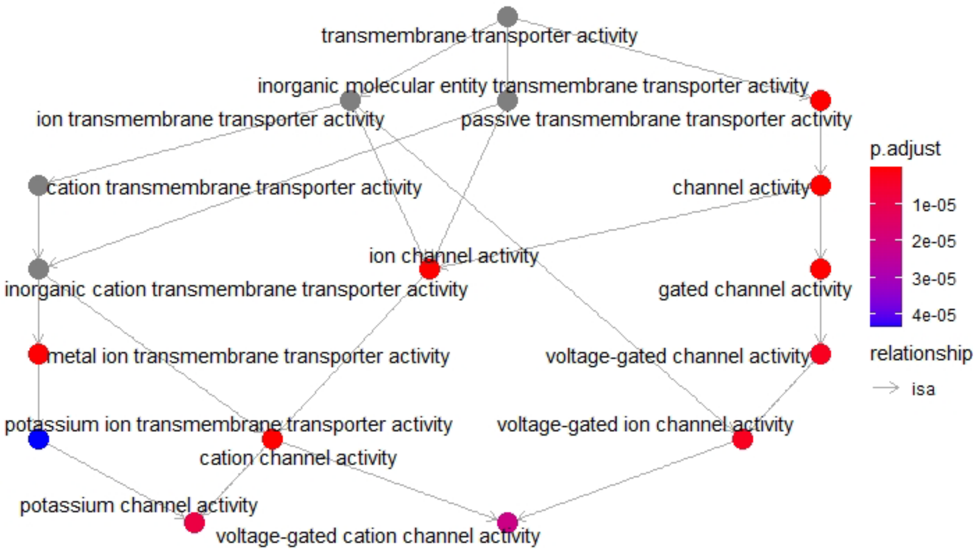

Figure S7: GO enrichment network plot of FV9 vs uninfected showing the most significant GO terms with their correspondent adjusted P-value. Differentially expressed genes in FV9-infected BMDMs compared with uninfected ones were extracted and analysed with ClusterProfiler.

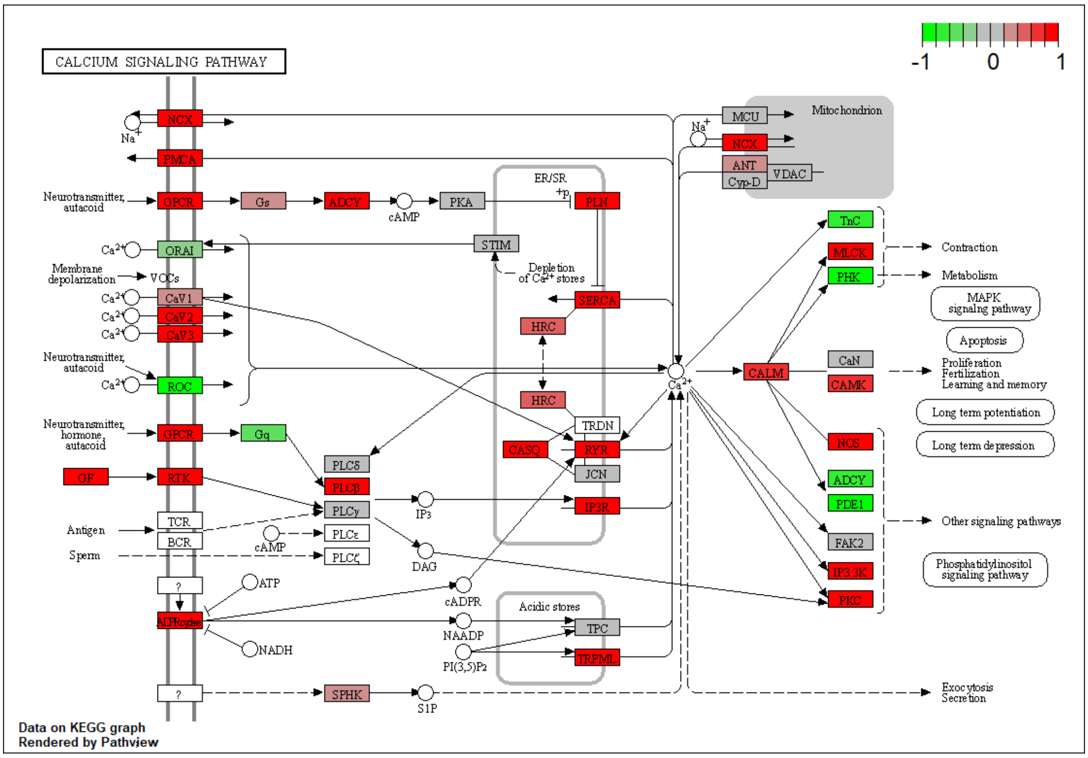

Figure S8: Calcium signalling pathway from KEGG map. Graphical representation showing DEGs in the Calcium signalling pathway leading to activation of PI3K-AKT or MAPK pathway induced by 5ASKH infection compared with uninfected BMDM. Upregulated genes are in Red, and downregulated are in green.

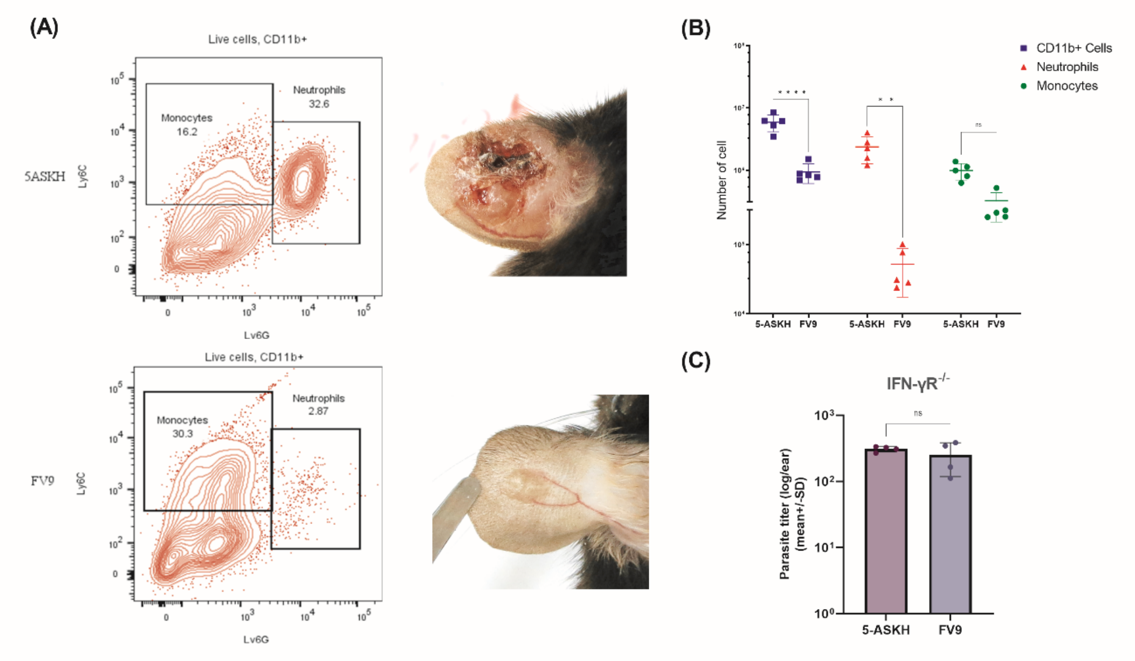

Figure S9: FV9 infection into IFN-γR$^{-/-}$ mice induced less pathogenesis with fewer neutrophil accumulation than 5-ASKH at 4 weeks post-infection.

Mice were intradermally IFN-γR$^{-/-}$ mice were infected with 5×10^4 5-ASKH or FV9 metacyclic promastigotes. At 4 weeks post-infection, ear tissues were processed and phenotyped by flow cytometry. **(A)** Representative ear photos with the corresponding flow cytometric dot plots of ear-infiltrating CD11b$^+$ myeloid cells, grouped as Ly6G^{high} Ly6C^{int} (neutrophils), Ly6G$^-$ Ly6C^{high} (monocyte). **(B)** The total number of cells recruited to the site of infection. **(B)** The corresponding parasite number in the infected ears.

Table S1: List of Antibodies and reagents used for Flow Cytometry.

**Table S1:** Reagent used for flow cytometric analysis				
**Antibody**	Clone	Catalogue Number	Manufacturer	Dilution Factor
**Zombie Yellow™ Fixable Viability Kit**		423104	Biolegend™	1/500 in PBS
**Zombie Aqua™ Fixable Viability Kit**		423101	Biolegend™	1/500 in PBS
**7-Amino-Actinomycin D (7-AAD)**		559925	BD Pharmingen™	5 µL/test
**TruStain FcX (anti-mouse CD16/32)**	93	101320	Biolegend™	1/500
**APC/Cyanine7 anti-mouse CD45 Antibody**	30-F11	103116	Biolegend™	1/400
**APC/Cyanine7 anti-mouse/human CD11b Antibody**	M1/70	101226	Biolegend™	1/400
**FITC anti-mouse/human CD11b Antibody**	M1/70	101206	Biolegend™	1/400
**PerCP-Cyanine5.5 CD11b Antibody**	M1/70	45-0112-80	ebioscience™	1/400
**APC anti-mouse Ly-6G Antibody**	1A8	127613	Biolegend™	1/300
**Biotin anti-mouse Ly-6G Antibody**	1A8	127603	Biolegend™	1/300
**Biotin anti-mouse Ly-6C Antibody**	HK1.4	128003	Biolegend™	1/200
**Ly-6C Monoclonal Antibody, Alexa Fluor 488**	HK1.4	53-5932-82	ebioscience™	1/200
**BV421 Rat Anti-Mouse CD49d**	9C10	740016	BD OptiBuild™	1/400
**PE anti-mouse CD284 (TLR4) Antibody**	SA15-21	145403	Biolegend™	1/400
**CD62L (L-Selectin) FITC,**	MEL-14	11-0621-82	ebioscience™	1/300
**APC anti-mouse F4/80 Antibody**	BM8	123116	Biolegend™	1/200
**Brilliant Violet 421™ anti-mouse F4/80 Antibody**	BM8	123137	Biolegend™	1/200
**PE anti-mouse CD206 (MMR) Antibody**	C068C2	141706	Biolegend™	1/300
**APC anti-mouse CD206 (MMR) Antibody**	C068C2	141708	Biolegend™	1/300
**MHC Class II (I-A/I-E) Monoclonal Antibody, PE**	M5/114.15.2	12-5321-82	ebioscience™	1/400
**APC Anti-Mouse MHC Class II (I-A/I-E)**	M5/114.15.2	20-5321	TONBO bioscience™	1/400
**PerCP-Cyanine5.5 Anti-Mouse MHC Class II (I-A/I-E)**	M5/114.15.2	65-5321	TONBO bioscience™	1/300
**APC anti-mouse CD38 Antibody**	90	102712	Biolegend™	1/400
**BV786 Hamster Anti-Mouse CD69**	H1.2F3	564683	BD Horizon™	1/400
**PerCP/Cyanine5.5 Streptavidin**		405214	Biolegend™	1/400
**Brilliant Stain Buffer**		563794	BD Horizon™	50 uL/test
**APC Mouse IgG1, κ Isotype Ctrl (FC) Antibody**	MOPC-21	400121	Biolegend™	1/300
**PE Mouse IgG2b, κ Isotype Ctrl Antibody**	MG2b-57	401207	Biolegend™	1/300
**CellCover**		800-125	ANACYTE™	
